# Time discrimination in psychosis: findings from a neuroimaging study

**DOI:** 10.1192/j.eurpsy.2023.335

**Published:** 2023-07-19

**Authors:** J. Goena, C. Vidal, S. Solís, M. Fernandez Seara, F. Ortuño, S. Garcés, M. Fernández

**Affiliations:** 1 Psychiatry; 2 Radiology, University of Navarra, Pamplona, Spain

## Abstract

**Introduction:**

Previous functional neuroimaging studies have demonstrated a brain network responsible of time discrimination (TD) processes, which may play a significant mediating role in other cognitive processes, such as change detection and cognitive control. The study of TD and its dysfunction in psychosis has become a matter of growing interest. We hypothesize that the impairment of the TD network is involved both in the mechanisms of psychosis and in the cognitive deficit presented by patients.

**Objectives:**

1. To delimit the brain regions involved in TD.
2. To examine the dysfunction in TD brain network in patients diagnosed with psychosis.
3. To sudy the integrity of brain white matter pathways in psychosis.
4. To verify whether the neuroimaging findings and TD test performance predict the neurocognitive profile of the patients.

**Methods:**

Participants included 20 patients with psychosis (PSY group) and 13 healthy controls (HC group). PSY group participants met remission criteria for 6 months prior to the study. Participants were interviewed for sociodemographic information and clinical assessments. They underwent a detailed cognitive assesment using the Measurement and Treatment Research to Improve Cognition in Schizophrenia (MATRICS) Consensus Cognitive Battery (MCCB). Neuroimaging study was performed on a 3 Tesla MRI scanner. We designed an experimental task including a test tool to assess TD and Oddbal detection (OD) paradigms with a cognitive control component. The task was conducted under functional magentic resonance imaging (fMRI). We used the general linear model analysis of the individual data of the fMRI images and the random effects model for group inference. Group differences in DTI were tested using tract-based spatial statistics (TBSS).

**Results:**

We find statistically significant differences (fMRI) in the activity related to TD (in HC), with greater activity in frontal cortical regions, the insular cortex and the cerebellum. In the PSY group, differences in the functionality and activation pattern of brain networks responsible for TD are observed, although voxel clustering does not reach the cluster significance limit when compared to HC. Compared to the HC, the PSY group has a significant deficit of fractional anisotropy (DTI) in the whole brain and in 21 specific brain regions. The PSY group has significantly lower scores in six of the seven cognitive domains than the control group, as well as in the overall composite. We correlated FA values in the groups of interest with MCCB scores.

**Image:**

**Figure:**
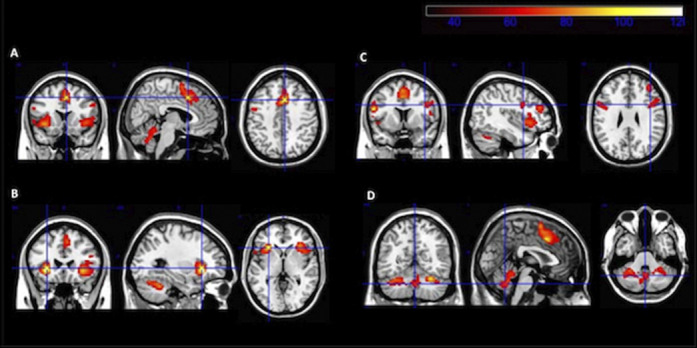

**Image 2:**

**Figure:**
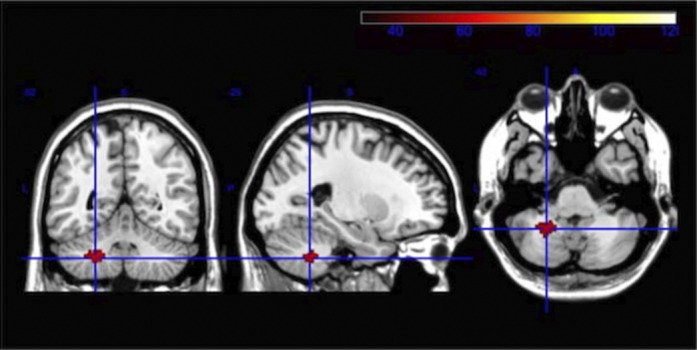

**Image 3:**

**Figure:**
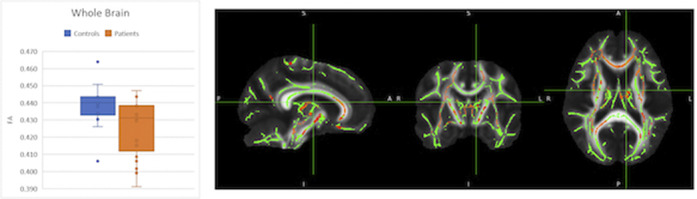

**Conclusions:**

We have defined the TD network, its relationship with other brain networks and cognitive processes of more complexity. The inclusion of participants with stable psychosis allowed us to analyze de TD disfunction in the PSY group. We compared the integrity of TD related brain pathways and correlated the findings with various clinical characteristics and the cognitive impairment present in psychotic patients.

**Disclosure of Interest:**

None Declared

